# Effects of prescribed antithrombotics and other cardiovascular pharmacotherapies on all-cause mortality in patients with diabetes and atrial fibrillation – a cohort study from Sweden using propensity score analyses

**DOI:** 10.1186/1758-5996-6-2

**Published:** 2014-01-07

**Authors:** Per Wändell, Axel C Carlsson, Jan Sundquist, Sven-Erik Johansson, Matteo Bottai, Kristina Sundquist

**Affiliations:** 1 Centre for Family Medicine, Karolinska Institutet, Alfred Nobels Allé 12, S-141 83 Huddinge, Sweden; 2 Geriatrics, Department of Public Health and Caring Sciences, Uppsala University, Uppsala, Sweden; 3 Center for Primary Health Care Research, Lund University/Region Skåne, Malmö, Sweden; 4 Stanford Prevention Research Center, Stanford University School of Medicine, Palo Alto, CA, USA; 5 Unit of Biostatistics, Institute of Environmental Medicine, Karolinska Institutet, Stockholm, Sweden

**Keywords:** Antithrombotic drugs, Statins, Pharmacotherapy, Mortality, Follow-up

## Abstract

**Aims:**

To study mortality rates among patients with diabetes and concomitant atrial fibrillation (AF), prescribed different cardiovascular drugs in primary health care.

**Methods:**

Study population consisted of men (n = 1319) and women (n = 1094) aged ≥45 years from a database including 75 primary care centres in Sweden. Cox regression analysis, with hazard ratios (HRs), 95% confidence interval (95% CIs) and mortality (years to death) as outcome, and Laplace regression, with difference in time to first 10% mortality (with 95% CI), were performed. Independent variables were prescribed cardiovascular drugs. Regression models were adjusted for a propensity score calculated separately for each prescribed drug class (comprising age, cardiovascular co-morbidities, education, marital status and pharmacotherapy).

**Results:**

Overall mortality was lower in the whole sample for anticoagulants vs no treatment (HR 0.45; 95% CI 0.26-0.77); and among patients < 80 years for anticoagulants vs. antiplatelets (HR 0.44; 95% CI 0.25-0.78); while among individuals aged ≥80 years, antiplatelets (HR 0.47; 95% CI 0.26-0.87) and anticoagulants (HR 0.49; 95% CI 0.24-1.00) vs. no treatment were equally effective. Statins were associated with lower mortality among those <80 years (HR 0.45; 95% CI 0.29-0.71). Laplace regression models in the whole sample, with years to first 10% of total mortality as outcome, were significant for: among patients < 80 years anticoagulants vs. no treatment 2.70 years (95% CI 0.04-5.37), anticoagulants vs. antiplatelets 2.31 years (95% CI 0.84-3.79), and those ≥80 antiplatelets vs. no treatment 1.78 years (95% CI 1.04-2.52).

**Conclusions:**

Our findings suggest that antiplatelets could exert a beneficial effect among those above 80 years.

## Introduction

Diabetes is a chronic disease affecting around 4.5% of the Swedish population [1]. Diabetes is associated with a 34% higher risk of developing atrial fibrillation (AF), the mechanisms of which are currently unclear [2].

The overall lifetime risk of developing AF from the age of 40 years has been estimated at 25%, irrespective of sex [3]. However, previous studies have shown a 1.5-fold greater risk of AF among men than among women [4], and that men develop AF five years earlier than women [5].

In contrast, women with AF have a higher excess mortality risk than in men with AF [6], and women also have a higher risk of stroke and different types of arrhythmias when prescribed antiarrhythmic drugs than do men [6].

The risk of stroke is markedly increased in AF patients, and anticoagulants are preferred to antiplatelets due to their superiority in stroke prevention [7]. However, anticoagulants are also effective in preventing myocardial infarctions [8], although their effect on mortality has not been adequately addressed [9]. As regards vascular and all-cause mortality, antiplatelets are just as effective as anticoagulants according to a Cochrane review [10].

In terms of pharmacological treatment and prevention of macrovascular complications in patients with diabetes, multifactorial treatment has been found to be effective [11], in particular treatment with statins [12], and with anti-hypertensive drugs [13]. It would be of interest to investigate whether these treatment strategies would be effective in reducing mortality in diabetic patients with AF, and if there are any gender and age differences. We have earlier described changes between 2002 and 2007 in the drugs prescribed to AF patients in primary care, with increased use of selective beta blockers, anticoagulant therapy and lipid-lowering [14]. Moreover, we have also found different effects of cardio-vascular drug classes on all-cause mortality in AF patients, with positive effects by statins among men and women < 80 years, anticoagulants among men and women, but not in women ≥ 80 years, and by antiplatelets among men ≥ 80 years [15]. Interestingly, an increased mortality was found among men < 80 years prescribed digitalis.

The primary aim of the present study was to compare the effects of antiplatelets and anticoagulants on mortality in men and women with diabetes and AF. A further aim was to understand the effects on mortality of cardiovascular drugs that are commonly prescribed to these patients.

## Methods

### Design

The study used individual-level patient data from 75 primary health care centers (PHCCs), 48 of which were located in Stockholm County. Individuals attending any of the participating PHCCs between 2001 and 2008 were included in the study. We used *Extractor* software (http://www.slso.sll.se/SLPOtemplates/SLPOPage1____10400.aspx; accessed September 19, 2010) to extract individual electronic patient records (EPRs). National identification numbers were replaced with new unique serial numbers to ensure anonymity. The files were linked to a database constructed using the Total Population Register, the Inpatient Register and the Swedish Cause of Death Register [16], which contains individual-level data on age, gender, education and hospital admissions for all residents registered in Sweden. Thus, a new research database containing clinical data and information on socioeconomic status on the 1,098,420 primary care-seeking individuals registered at the 75 PHCCs was created. Data from the Cause of Death Register, which has been shown to be 99.8% complete [17], were used for the follow-up.

Ethical approvals were obtained from regional ethical boards at Karolinska Institutet and the Lund University.

### Study population

All persons diagnosed with both diabetes and AF who attended the 75 PHCCs between January 1^st^ 2001 and December 31^st^ 2007 were included in this study. They were identified by the presence of the ICD-10 (10th version of the WHO’s International Classification of Diseases) codes for AF (I48) and diabetes mellitus (E10-14) in the medical records. In total, 2,413 individuals, 1,319 men and 1,094 women, who were ≥45 years of age at the time of AF diagnosis were identified and included [18].

### Outcome variable

Time to death in the period from registration of AF diagnoses until December 31^st^ 2007.

### Pharmacotherapies

Drugs prescribed to individuals in the study population during the assessment period were recorded by Anatomic Therapeutic Chemical (ATC) Classification. With regard to anti-thrombotic drugs (B01A), patients were divided into three groups: no treatment, anti-platelet treatment with no anticoagulant treatment (B01AC), including acetylsalicylic acid (ASA; B01AC6, B01AC30), anticoagulant treatment with no anti-platelet treatment (B01AA), and finally a group who had received both anticoagulant and anti-platelet treatment. Diuretic drugs (C03) were recorded as thiazides or related agents, and were also registered when in combination with other drugs (C03A, C03B, C03E, C09B C09DA), loop diuretics (C03C) or potassium-saving diuretics (C03D), including aldosterone antagonists (C03DA). Furthermore, the following cardiovascular agents were recorded: heart-active drugs (C01), beta blockers (C07), calcium receptor-blocking agents (C08), and RAS-blocking agents (C09). Lipid-lowering drugs (C10A), including statins (C10AA), were also recorded.

### Demographic and socio-economic variables

*Gender:* Men and women.

Individuals were divided into the following *age groups* 45–54, 55–64, 65–74, 75–84 and ≥85 years. Individuals younger than 45 years were excluded.

*Educational level* was categorized as ≤9 years (partial or complete compulsory schooling), 10–12 years (partial or complete secondary schooling) and >12 years (college and/or university studies).

*Marital status* was classified as married, unmarried, divorced or widowed.

### Co-morbidities

We identified the following cardiovascular co-morbidities from the EPRs among individuals in the study population: hypertension (I10-15), coronary heart disease (CHD; I20-25), heart failure (HF; I50 and I110), non-rheumatic valvular diseases (I34-38), cardiomyopathy (I42) and cerebrovascular diseases (CVDs; I60-69), including intracranial bleeding (I60-62).

### Statistical analyses

Differences in means and distributions between men and women were compared by Student’s *t*-test, chi-square analysis and Fisher’s exact test.

Follow-up analyses were performed using Cox regression with hazard ratios (HRs) and 95% confidence interval (95% CI), using time to death as the outcome. Laplace regression was used to calculate the difference in years until any given percentage of the participants prescribed pharmacotherapies vs. those without these particular drugs die [19,20]. We calculated the difference in years until 10% of the participants died for those prescribed a particular therapy vs. those not prescribed. The 10^th^ percentile was chosen, as the results are more reliable in Laplace regression if both exposed and unexposed individuals have a sufficient number of fatalities (events) during the follow-up. Laplace regression also uses bootstrap technique in the estimation of confidence intervals. Since different distributions and mathematical calculations are used to obtain results in Cox and Laplace regression, respectively, putting emphasis on findings significant with both methods reduces the risk of chance findings.

Models were tested for interaction between sex and each pharmacotherapy. Analyses were performed for individuals <80 and ≥80 years of age. 80 years has commonly been referred to as a cut-off age for stroke prophylaxis [21], as well as the beginning of a period of increased prevalence and incidence of AF [22]. Furthermore, there were too few women aged <75 when they died (n = 18) to provide reliable results.

In the regression models, prescription of antithrombotic drugs (anti-platelets or anticoagulants), digitalis, diuretics (thiazides, loop diuretics and aldosterone antagonists), beta blockers, RAS-blocking agents, calcium receptor-blocking agents and statins were used as independent factors in the analyses. For antithrombotic drugs we used three groups: no antithrombotic treatment, anti-platelets only and anticoagulants only. We excluded the group with both anti-platelets and anticoagulants, as it would be hard to interpret the effects seen. We excluded specific anti-arrhythmic agents, potassium-saving agents other than aldosterone antagonists and lipid-lowering agents other than statins due to their low prescription rates. We did not divide RAS-blocking agents into ACE inhibitors and ARB, or calcium receptor-blockers into those with vessel- and heart-specific effects. Adjustment was made for a propensity score comprising age group, cardiovascular co-morbidities (hypertension, CHD, HF and CVDs), educational level, marital status, and all pharmacotherapies [23]. A unique propensity score was constructed for each drug class to balance all other cardiovascular drug classes, as well as co-morbidities, age, sex (in sex-adjusted models) and socio-economic factors. We excluded valvular diseases and cardiomyopathy from the analyses due to the low numbers of cases.

A *p*-value for two-sided tests of <0.01 was considered statistically significant. This cut-off was used due to the multiple comparisons between men and women. A *p*-value of <0.05 was considered statistically significant for variables in the Cox and Laplace regression analyses, using 95% confidence intervals.

## Results

Characteristics of the study population (n = 2,413) are shown separately for men (n = 1319) and women (n = 1094) in Table 1. A total of 120 men (9.1%) and 145 women (13.3%) died during follow-up (*p* = 0.0012). Overall, men were significantly younger and had more education than did women, while significantly more women were widowed than were men. HF and valvular disease were more common among women, while CVDs were more common among men. CHD and hypertension were more common among women than men (Table 1). The mean follow-up time was 3.7 years (standard deviation (SD) 2.1) and HRs were calculated based on 8,999 person-years at risk.

**Table 1:** **Baseline data for patients aged ≥45 years with diagnoses of AF and diabetes mellitus (*n*** **= 2,413) in primary care attending the 75 PHCCs between January 1**^**st **^**2001 and December 31**^**st **^**2007**

	**Men n = 1,319**		**Women n = 1,094**		**Sex difference**	
	**All**	**Dead**	**All**	**Dead**	**All**	**Dead**
	***n *** **(%)**	***n *** **(%)**	***n *** **(%)**	***n *** **(%)**	***p***	***p***
Number of patients	1,319	120 (9.1)	1,094	145 (13.3)		0.0012
Age (years), mean (SD)	71.7 (8.8)		76.3 (8.5)		<0.001	
Age (years),					<0.001	
45–54	36 (2.7)	3 (8.3)	11 (1.1)	0		0.32
55–64	257 (19.5)	8 (3.1)	102 (9.3)	5 (4.9)		0.41
65–74	494 (37.5)	26 (5.3)	293 (26.8)	13 (4.4)		0.61
75–79	265 (20.1)	27 (10.2)	246 (22.5)	30 (12.2)		0.47
80–84	184 (14.0)	37 (20.1)	271 (24.8)	47 (17.3)		0.46
≥85	83 (6.3)	19 (22.9)	171 (15.6)	50 (29.2)		0.29
Educational level					<0.001	
Compulsory schooling	559 (43.9)	45 (8.1)	582 (59.4)	76 (13.1)		0.0060
Secondary schooling	478 (37.5)	42 (8.8)	309 (31.5)	25 (8.1)		0.73
College and/or university studies	237 (18.6)	21 (8.9)	89 (9.1)	7 (7.9)		0.78
Marital status					<0.001	
Married	742 (46.5)	63 (8.5)	317 (29.1)	32 (10.1)		0.40
Unmarried	148 (11.3)	9 (6.1)	76 (7.0)	10 (13.2)		0.072
Divorced	229 (17.4)	17 (7.4)	151 (13.9)	15 (9.9)		0.39
Widowed	194 (14.8)	30 (15.5)	545 (50.1)	85 (15.6)		0.97
Diagnosis						
Hypertension	789 (59.8)	53 (6.7)	677 (61.9)	70 (10.3)	0.30	0.013
Coronary heart disease	357 (27.1)	28 (7.8)	285 (26.1)	46 (16.1)	0.57	0.001
Chronic heart failure	228 (17.3)	38 (16.7)	250 (22.9)	46 (18.4)	0.001	0.62
Valvular disease	50 (3.8)	6 (12.0)	62 (5.7)	14 (22.6)	0.029	0.15
Cardiomyopathy	15 (1.1)	1 (6.7)	9 (0.8)	1 (11.1)	0.44	0.62
Cerebrovascular diseases	187 (14.2)	15 (8.0)	121 (11.1)	18 (14.9)	0.022	0.057

Table 2 shows rates of prescription of cardiovascular pharmacotherapies in men and women. Greater proportions of men were prescribed antithrombotic drugs, anticoagulants, specific anti-arrhythmic agents, RAS-blocking agents, ACE inhibitors, lipid-lowering drugs and statins. Greater proportions of women were prescribed digitalis, diuretics, loop diuretics, potassium-saving agents, beta blockers and beta1-selective agents. There were no gender differences in the total number of prescribed cardiovascular drugs.

**Table 2:** **Prescription of drug classes for patients aged ≥45 years with diagnoses of AF and diabetes mellitus (*n*** **= 2,413) in primary care attending the 75 PHCCs between January 1**^**st **^**2001 and December 31**^**st **^**2007**

	**Men n = 1,319**	**Women n = 1,094**	**Sex difference**
	***n *** **(%)**	***n *** **(%)**	***p***
All antithrombotic agents (B01A)	1,223 (92.7)	978 (89.4)	0.005
Anticoagulant agents (B01AA)	806 (61.1)	567 (51.8)	<0.001
Antiplatelet agents (B01AC)	822 (62.3)	688 (62.9)	0.77
ASA (B01AC6, B01AC30)	800 (60.7)	672 (61.4)	0.70
Antithrombotic treatment group:			
No antithrombotic agent	96 (7.3)	116 (10.6)	0.004
Antiplatelets only	417 (31.6)	410 (37.5)	0.003
Anticoagulants only	401 (30.4)	290 (26.5)	0.035
Both antiplatelets and anticoagulants	405 (30.7)	278 (25.4)	0.004
Digitalis (C01A)	521 (39.5)	596 (54.5)	<0.001
Specific anti-arrhythmic agents (C01B)	40 (3.0)	17 (1.6)	0.017
Any diuretic treatment (C03)	972 (73.7)	916 (83.7)	<0.001
Loop diuretics (C03C)	794 (60.2)	772 (70.6)	<0.001
Thiazides (C03A, C03B, C03E, C09B C09DA)	357 (27.1)	334 (30.5)	0.061
Any potassium-saving agent (C03D)	414 (31.4)	420 (38.4)	<0.001
Aldosterone antagonists (C03DA)	276 (20.9)	264 (24.1)	0.060
All beta blockers (C07)	1,008 (76.4)	845 (77.2)	0.64
Beta1-selective agents (C07AB, C07F)	873 (66.2)	769 (70.3)	0.031
Non-selective beta blockers (C07AA)	276 (20.9)	203 (18.6)	0.15
Calcium receptor-blocking agents (C08)	535 (40.6)	452 (41.3)	0.071
RAS-blocking agents (C09)	952 (72.2)	708 (64.7)	<0.001
ACE inhibitors (C09A, C09B)	776 (58.8)	519 (47.4)	<0.001
ARB (C09C, C09D)	352 (26.7)	324 (29.6)	0.11
All lipid-lowering drugs (C10A)	670 (50.8)	459 (42.0)	<0.001
Statins (C10AA)	650 (49.3)	452 (41.3)	<0.001
Number of cardiovascular drugs			0.53
0	16 (1.2)	19 (1.7)	
1	36 (2.7)	18 (1.7)	
2	69 (5.2)	59 (5.4)	
3	121 (9.2)	82 (7.5)	
4	196 (14.9)	158 (14.4)	
5	264 (20.0)	215 (19.7)	
6	274 (20.8)	226 (20.7)	
7	189 (14.3)	182 (16.6)	
8	107 (8.1)	91 (8.3)	
9	36 (2.7)	36 (3.3)	
10	10 (0.8)	8 (0.7)	
11	1 (0.0)	0 (0.0)	

Prescription is noted at any time during the time period.

Table 3 and Table 4 show Cox regression and Laplace regression models stratified by age into two groups, <80 years and ≥80 years, respectively. Significantly lower HRs of mortality (Table 3) and longer survival (in years; Table 4) for anticoagulants vs no treatment and for anticoagulants vs antiplatelets in patients <80 years of age were detected. Significantly lower HRs of mortality (Table 3) and longer survival (in years; Table 4) were found for antiplatelets vs no treatment in patients aged ≥80 years. Diverse effects were seen in individuals ≥80 years; significantly lower risks in Cox regression but not in Laplace regression was detected for anticoagulants vs no treatment. Significant interactions in Cox regression models as regards anticoagulant treatment and high or low age group were found (p = 0.001). We also tested for effects in the different age groups, and antiplatelets vs no treatment showed lower estimates in Cox regression models in the ages 80–84 and 85–96 years (0.51 and 0.41, respectively) but not in the ages 65–79 years (data not shown), while anticoagulants vs antiplatelets showed significantly lower estimates in the ages below 75 years (HR 0.33, 95% CI 0.14-0.80). Significantly low HRs of mortality (Table 3) and longer survival (Table 4) was also found for beta blockers and for statins in in patients <80 years of age, and also in models with all patients.

**Table 3:** **Cox regression models with total mortality as outcome for patients for patients aged ≥45 years with diagnoses of AF and diabetes mellitus (*n*** **= 2,413) attending the 75 PHCCs between January 1**^**st **^**2001 and December 31**^**st **^**2007, stratified by age: 45–79 years (n = 1,704) and 80–104 years (n = 709)**

	**Patients aged <80 years**	**Patients aged ≥80 years**	**All patients**
	**Full model**	**Full model**	**Full model**
	**HR (95% CI)**	**HR (95% CI)**	**HR (95% CI)**
Antithrombotic drugs			
Antiplatelets vs. no treatment	0.86 (0.43-1.72)	**0.47 (0.26-0.87)**	0.70 (0.45-1.09)
Anticoagulants vs. no treatment	**0.40 (0.17-0.91)**	**0.49 (0.24-1.00)**	**0.45 (0.26-0.77)**
Anticoagulants vs. antiplatelets	**0.44 (0.25-0.78)**	1.02 (0.60-1.73)	**0.62 (0.42-0.92)**
Digitalis	0.81 (0.54-1.23)	0.90 (0.60-1.35)	0.83 (0.62-1.11)
Loop diuretics	**2.42 (1.36-4.33)**	1.02 (0.60-1.73)	**1.55 (1.06-2.28)**
Thiazides	0.98 (0.59-1.63)	0.89 (0.55-1.44)	0.93 (0.66-1.32)
Aldosterone antagonists	1.20 (0.76-1.88)	1.22 (0.79-1.88)	1.29 (0.94-1.77)
Beta blockers	**0.62 (0.40-0.97)**	0.73 (0.49-1.10)	**0.67 (0.49-0.90)**
RAS-blocking agents	1.08 (0.65-1.78)	1.23 (0.79-1.93)	1.16 (0.83-1.62)
Calcium receptor-blocking agents	1.06 (0.69-1.63)	1.20 (0.77-1.87)	1.10 (0.81-1.49)
Statins	**0.45 (0.29-0.71)**	0.77 (0.47-1.26)	**0.56 (0.40-0.78)**

Full model is adjusted for a propensity score comprising sex, age group, co-morbidities, educational level, marital status and for all pharmacotherapies. HRs and 95% CIs are shown. Statistically significant HRs are highlighted in bold.

**Table 4:** **Laplace regression models with years to first 10% of total mortality as outcome for patients aged ≥45 years with diagnoses of AF and diabetes mellitus (*n*** **= 2,413) attending the 75 PHCCs between January 1**^**st **^**2001 and December 31**^**st **^**2007, stratified by age: 45–79 years (n = 1,704) and 80–104 years (n = 709)**

	**Patients aged <80 years**	**Patients aged ≥80 years**	**All patients**
	**Full model**	**Full model**	**Full model**
	**HR (95% CI)**	**HR (95% CI)**	**HR (95% CI)**
Antithrombotic drugs			
Antiplatelets vs. no treatment	0.81 (-1.24; 2.87)	**1.78 (1.04; 2.52)**	1.02 (-0.34; 2.39)
Anticoagulants vs. no treatment	**2.70 (0.04; 5.37)**	1.22 (-0.06; 2.51)	**2.20 (0.79; 3.62)**
Anticoagulants vs. antiplatelets	**2.31 (0.84; 3.79)**	0.23 (-1.10; 1.55)	1.21 (-0.03; 2.45)
Digitalis	1.02 (-0.26; 2.30)	0.25 (-2.35; 2.85)	0.58 (-0.28; 1.45)
Loop diuretics	**-2.44 (-3.79; -1.08)**	0.69 (-0.89; 2.26)	-1.22 (-2.79; 0.34)
Thiazides	0.18 (-1.34; 1.70)	**1.09 (0.10; 2.07)**	0.35 (-0.56; 1.24)
Aldosterone antagonists	-0.17 (-1.23; 0.88)	-0.17 (-0.89; 0.56)	-0.07 (-0.90; 0.76)
Beta blockers	1.28 (-0.13; 2.69)	0.29 (-0.47; 1.06)	**1.18 (0.32; 2.04)**
RAS-blocking agents	-0.18 (-1.40; 1.04)	-0.19 (-0.74 (0.36)	0.23 (-0.57; 1.02)
Calcium receptor-blocking agents	-0.02 (-1.20; 1.16)	-0.07 (-0.86; 0.72)	-0.54 (-1.61; 0.53)
Statins	**2.11 (0.88; 3.35)**	0.38 (-1.37; 2.14)	**1.24 (0.38; 2.10)**

Full model is adjusted for a propensity score comprising sex, age group, co-morbidities, educational level, marital status and for all pharmacotherapies. HRs and 95% CIs are shown. Statistically significant HRs are highlighted in bold.

Significant interactions were found regarding sex and digitalis (p = 0.002), as well as sex and loop diuretics (p = 0.003). For digitalis, in men HR was 1.13 (95% CI 0.76-1.68) and in women 0.54 (95% CI 0.35-0.82), and for loop diuretics, in men HR was 2.51 (95% CI 1.39-4.53) and in women 0.91 (95% CI 0.54-1.53).

## Discussion

The main findings of this study was that anticoagulants were superior in reducing mortality among individuals with diabetes and AF below 80 years of age, while antiplatelet therapy seemed equally effective as anticoagulants among individuals 80 years and above. An unexpected finding was that mortality was reduced in women who were prescribed digitalis, but not in men. In addition, mortality was increased in men prescribed loop diuretics. Furthermore, we found that mortality was reduced in patients aged below 80 years prescribed statins, and also among those below 80 years prescribed beta blockers.

One might have expected that all prescribed pharmacotherapies would decrease mortality among the population in this study. However, the benefits of a drug could be offset by side effects, as in the case of disopyramide, quinidine and sotalol, which are known to be associated with increased mortality [24]. The studied subgroup of subjects, i.e. AF patients with diabetes, could show a risk pattern different to what could be expected in AF patients in general.

An interesting finding was the equally positive effects of antiplatelets and anticoagulants on mortality among individuals ≥80 years of age, making findings from a Cochrane review accountable also for patients with diabetes and AF [10]. Although anticoagulants are superior to antiplatelets in stroke prevention of AF patients [7], the current study indicates that among elderly individuals antiplatelets could be useful for those who do not tolerate anticoagulants because of equal effects on all-cause mortality.

Prescription of digitalis was associated with decreased mortality only among women. It seems, according to a review, that beneficial effects of digitalis are obtained at low digoxin concentrations, while high serum levels were associated with increased mortality [25]. Positive effects of digitalis in AF and CHF seem to be related to attenuation of sympathetic activation and neurohumoral alterations. Diabetes is associated with an impaired myocardial energy production affecting myocyte contraction and diastolic function [26], leading to diastolic heart dysfunction, and little is known about the optimal treatment of this condition [27]. The female myocardium could possibly benefit more from the effects of digitalis than the male. Another explanation could be that the gender differences might reflect different prescription patterns in men and women with AF. The previously reported higher mortality among men < 80 years [15] could be due to more pronounced CHF [28], and consequently higher digitalis doses.

The finding of an association between prescription of loop diuretics with an increased mortality among men could be explained by concomitant presence of CHF, as CHF in AF patients do increase mortality [29]. Prescribed loop diuretics may be a marker of more advanced CHF, but this is contradicted by the fact that other possible markers of this, i.e. digitalis [30], and aldosterone antagonists [31], were not associated with significantly increased mortality. Another possibility is that loop diuretics could have increased arrhythmia risks due to potassium depletion [32,33].

The low mortality associated with prescribed beta blockers was expected, considering the effect in reducing mortality in myocardial infarction with AF [34], and also in CHF [35,36].

The reduced mortality associated with prescription of statins is in line with evidence of positive effect of statins on diabetic patients [12]. The mortality reduction associated with statins was more pronounced compared to a meta-analysis of randomized trials [12], as is commonly the case in observational studies using propensity scores [23]. Statins have been shown to have anti-arrhythmic properties [37], which could be relevant for patients with AF. However, it cannot be excluded that the positive finding for survival could be due to confounding by indication, i.e., severely ill patients not being prescribed statins to the same extent.

Crude mortality was higher in women than in men, in agreement with others’ findings [38]. In an earlier Swedish study, we showed that men with AF had a relative mortality risk of 1.3 and women with AF a relative mortality risk of 1.9 when they were compared to men and women in the general population [39]. The women in this study were on average five years older than the men (mean age 76.3 vs. 71.7), and could consequently be expected to have 11% higher mortality rate than men (data from Statistics Sweden). However, we found a 46% higher mortality rate in women compared to men, which could be explained by the excess mortality risk in women due to both diabetes [40], as well as to AF [6], or the co-morbidity.

A limitation of this study was the small sample size, which prevented us from detecting small differences, even though the analyses included almost 9,000 person-years at risk. Since this was an observational study of the association between prescription of drugs and mortality, the findings may have been subject to confounding by indication [41,42]. Furthermore, drugs prescribed by other caregivers were not included in the patient records, which may have weakened the associations between prescription of certain medications and mortality in this study. We did not have access to doses of the prescribed drugs. Severity of CHF and CHD were not classified in the patient records. Moreover, AF could not be classified as paroxysmal, persistent or permanent and heart rhythm could not be classified as sinus rhythm or fibrillation rhythm. We had not access to data on renal disease, and besides not of non-cardiovascular diseases or pharmacotherapies. Thus, we cannot exclude the possibility that residual confounding may explain at least partly some of the results.

A major strength of this study was that we were able to link clinical data from individual EPRs to data from national demographic and socioeconomic registers with less than 1% of information missing. In addition, an earlier study of the diagnoses recorded in the EPRs showed that less than 2% of all diagnoses per individual were missing [43]. The use of a propensity score with covariates relevant to mortality as the outcome was an additional strength of this study as it enabled adjustment with a low risk of over-fitting regression models [44]. Besides, we used Laplace regression to confirm results of Cox regression in the entire sample.

In conclusion, we found significantly positive effects of both antiplatelets and anticoagulants on survival among individuals aged ≥80 years, which could imply that antiplatelets despite little effect in stroke prevention could be beneficial on survival in elderly diabetes patients with AF when anticoagulants cannot be prescribed. We also found gender differences, indicating a positive effect by digitalis on survival among women, and a negative effect by loop diuretics among men, both needing further attention. The decreased mortality risk associated with prescription of statins underlines the positive effect of statins in diabetes patients with AF, and treatment by statins could be as important as antithrombotics in these patients. More studies focusing on diabetes patients with AF are needed to confirm or reject our findings.

## Competing interest

The authors declare that they have no competing interest to disclose.

## Authors’ contribution

JS and KS are the principal investigators of the database study, and contributed in the drafting of the manuscript. PW and ACC brought the idea of the specific study, PW performed the statistical analyses in discussion with ACC, PW drafted the manuscript in discussion with ACC. SEJ and MB contributed to the statistical analyses and to discussion of the statistical findings, MB especially on the Laplace regression method. All authors read and approved the final manuscript.
